# Supplementary material for: Mitogen-Activated Protein Kinase (MAPK) and Obesity-Related Cancer
Source: Int J Mol Sci. 2020 Feb 13;21(4):1241. doi: 10.3390/ijms21041241 (PMC7072904; doi:10.3390/ijms21041241)
Supplement: Supplementary file 1 [file ijms-21-01241-s001.pdf]

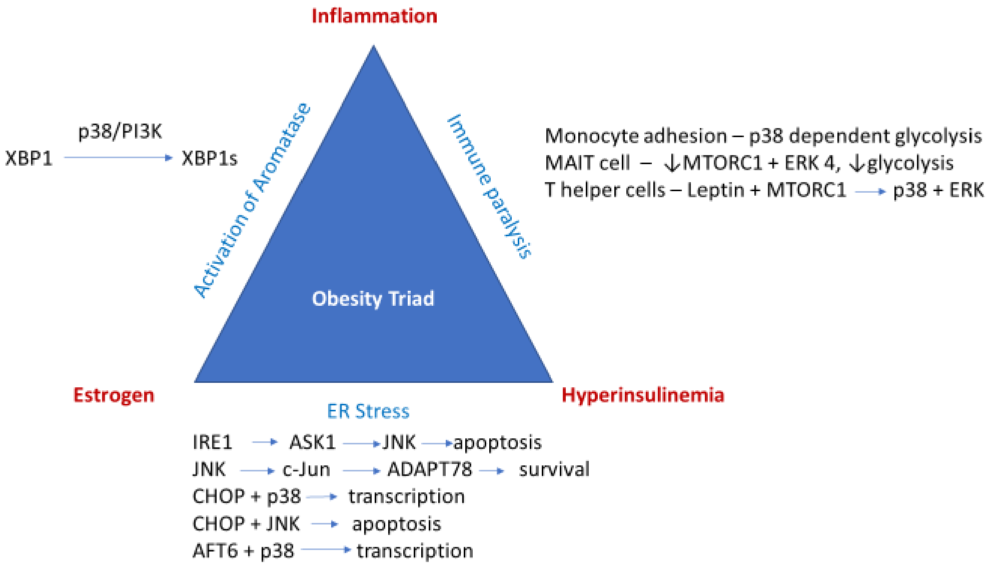

Figure S1. Interaction between Obesity triad and MAPK signalling. Obesity is associated with the triad of inflammation, hyperinuslinemia and increased oestrogen signalling, with associated outcomes including increased aromatisation, ER stress and immune paralysis. There is increasing evidence to support different types of MAPK signalling in many of these processes.
